# Efficacy of yoga-breath linked intervention to stop smoking (Y-BLISS) in reducing the number of cigarettes smoked per day among smokers attempting to reduce or quit: Study protocol for a randomized controlled trial

**DOI:** 10.12688/wellcomeopenres.22839.1

**Published:** 2024-11-26

**Authors:** Chandra Shekar MV, Hemant Bhargav, Bharath Holla, Jayant Mahadevan, Amit Singh, Venkata Lakshmi Narasimha, Naresh Katla, Atmakur Snigdha, Komal S, E Maheswari

**Affiliations:** 1 Yoga and Life Sciences, Swami Vivekananda Yoga Anusandhana Samsthana (S-VYASA)), Bengaluru, Karnataka, 560019, India; 2 Intergrative Medicine, National Institute of Mental Health and Neurosciences (NIMHANS)), Bengaluru, Karnataka, 560029, India; 3 Centre for Addiction Medicine, Department of Psychiatry, National Institute of Mental Health and Neurosciences (NIMHANS), Bengaluru, Karnataka, 560029, India; 4 Yoga and science of living, Jain Vishva Bharathi Institute, Ladnun, Rajasthan, 341306, India; 5 Departmental pharmacy, M.S. Ramaiah University of applied sciences, Bengaluru, Karnataka, 560054, India

**Keywords:** Yoga, Exercise, Smoking, Addiction, Cravings

## Abstract

**Background:**

The proposed research aims to test the effects of a two-month yoga-breath linked intervention to stop smoking (Y-BLISS) as an add-on to standard treatment in tobacco use disorder (TUD; in the form of cigarette smoking) by conducting a randomized controlled study with the following primary outcome variable: number of cigarettes smoked per day. Secondary outcome variables are motivation to stop smoking, craving, dependence severity, lung capacity, and exhaled carbon-monoxide.

In this single-blinded, randomized, controlled, parallel-group superiority trial with 1:1 allocation ratio, 100 patients with TUD availing the outpatient/ inpatient clinical services at a tertiary mental healthcare hospital in India will be enrolled after giving informed consent. Consecutive consenting patients will be randomly allotted to one of the two groups – yoga arm (Y-bliss arm), or desktop exercise arm (D-exercise arm). Allocation concealment will be followed, the clinicians, outcome assessors and data analysts will remain blind to subject-group allocation. A validated and standardized 12-minute yoga program (Y-BLISS) for TUD will be used as an intervention. The control group will receive an equivalent 12-minute desktop exercise module. Participants in respective arms will receive 40 online supervised sessions over 8 weeks, 5 sessions per week in the mornings as they will be asked to practice the same on sos basis whenever they feel cravings for cigarette smoking. Assessments will be done at baseline, 4th week and 8thweek. Data from all randomized subjects will be analyzed using intent-to-treat analysis and mixed model multivariate analysis.

**Dissemination:**

Findings will be disseminated through peer-reviewed publications, conference presentations, and social media.

**Trial registration number:**

The trial has been registered under Clinical Trials Registry India (CTRI) with the registration number: CTRI/2022/12/048117, date of registration: 14 ^th^ December 2022.

## Plain language summary

This article provides the study protocol for a randomized controlled trial where we aim to compare the effect of add-on yoga with add-on exercise on the severity of addiction in people (n = 100) who are cigarette smoking dependent and who wish to quit. We aim to provide an intervention duration of 8 weeks and perform clinical assessments along with some objective measures to assess lung functions and exhaled carbon monoxide at the following time points: baseline, week 4, and week 8.

## Background

Tobacco smoking is a worldwide epidemic with more than one billion consumers. Tobacco consumption is one of the commonest addictions and is a leading cause of morbidity and mortality worldwide. The World Health Organization estimates 8 million casualties every year resulting from tobacco consumption ( Reitsma *et al.*, 2021). The most prevalent, and widely researched form of tobacco consumption is cigarette smoking. In India, 19 percent of the male, and 2 percent the female adult population smoked tobacco as per survey done in the year 2016–2017 ( GATS, 2016). More recent National Family Health Survey ( NFHS 5, 2021) found an overall prevalence of tobacco use to be 40.3% (39% in men; 4% in women) with 13.3% of them smoking cigarettes. Cigarette smoking is reported to be the cause of premature death in every one out of two dependent users. About half of all tobacco-related deaths occur between the ages of 35 and 69, resulting in a loss of 20 to 25 years of life expectancy compared to non-smokers ( Jha *et al.*, 2006). These adverse effects have been found in both male and female populations. Research has shown that females are more susceptible to smoking-related morbidity and mortality ( Allen *et al.*, 2014)

The Government of India enacted the 'Cigarettes and Other Tobacco Products (Prohibition of Advertisement and Regulation of Trade and Commerce, Production, Supply and Distribution) Act (COTPA) in 2003, and revised in 2008. The aim of the Act was to prohibit the consumption of cigarettes and other tobacco products, which are injurious to health. The revision in 2008 focused on redefining 'public places' to include all workplaces and authorising personnel responsible for enforcement of the law for maintaining smoke - free public places across the country ( Gupta *et al.*, 2024).

Conventionally, both pharmacological and non-pharmacological approaches are helpful in improving smoking abstinence. Mainstay of pharmacotherapy is nicotine replacement therapy (NRT) in the form of nicotine patch, gum, lozenge, nasal spray and oral inhaler. Non-nicotine medications such as Bupropion, Varenicline tartrate are the other useful options. Non-pharmacological interventions with proven utility in smoking cessation include behavioural therapies such as counselling, support groups, telephone quit-line, physical exercises, and mindfulness approaches ( Jha & Li, 2017). Pharmacological therapies though effective in reducing craving, are costly and the non-pharmacological approaches are not easily accessible especially in country like India where there is a large gap between the demand and supply of trained human resource in psychotherapy. Moreover, a need for culturally sensitive interventions has been emphasized. Smoking cessation can be very challenging. A study showed that at any given point of time 70% of smokers had the desire to quit and 46% had attempted to quit but were not able to maintain abstinence. Over 50% of these subjects had relapsed in the past one year ( Koçak *et al.*, 2015). Psychosocial stressors increase the chances of relapse following a quit attempt ( Shiffman *et al.*, 1996). Study showed that smokers who failed to quit or relapsed after a short period reported high levels of stress prior to quitting after cessation. On the other hand, participants who were able to quit and maintain abstinence at 6-month follow-up reported a gradual decrease in stress levels ( Cohen & Lichtenstein, 1990).

Considering the above, there is a need for a low cost, scalable, culturally more acceptable intervention that can generate a feeling of self-empowerment in the practitioner. One such initiative in India is the use of digital technology, exemplified by the NIMHANS Tobacco Quitline Services (NTQLS) implemented by the National Institute of Mental Health and Neurosciences, Bangalore ( Pradeepkumar *et al.*, 2023). Another promising approach could involve leveraging traditional therapies, such as yoga.Yoga and meditation are increasingly recognized for their ability to enhance recovery from addiction by targeting stress-related cognitions, emotions, and behavioral urges such as craving. The insights and self-awareness learned through yoga practice (which differentiate it from exercise can target multiple psychological, physiological, and behavioral processes that are implicated in addiction and relapse ( Khanna & Greeson, 2013). While exploring novel interventions is important, efforts should also focus on increasing the number of tobacco cessation centers in India ( Narasimha *et al.*, 2021). These centers can play a crucial role in delivering such interventions effectively at the community level.

In light of the above, a systematic research plan to understand role of yoga-breath linked intervention to stop smoking (Y-BLISS) as an adjuvant in the management of cigarette smoking dependence is proposed.

## Objectives

### Primary objectives

To evaluate the effect of a two-month add-on Y-BLISS program on number of cigarettes smoked per day in participants with nicotine dependence in the form of smoking cigarettes.

### Secondary objectives

To evaluate the effect of a two-month add-on Y-BLISS program as compared to an equivalent desktop exercise program (D-exercise) on

1. motivation to stop smoking using Motivation to Stop (MTSS) Scale

2. craving of smoking using tobacco craving questionnaire

3. dependence severity using FTND

4. exhaled carbon monoxide [Exhaled CO] using Bedfont Breath Analyzer

5. lung capacity using peak flow meter (liters/minute)

### Hypothesis

The Y-BLISS program will serve as a useful adjunct therapy to standard treatment as compared to D-exercise program in participants with cigarette smoking dependence in

Primary-1) reducing number of cigarettes smoked per day

Secondary-1) improving motivation to stop smoking, 2) reducing craving, 3) reducing dependence, 4) improving lung capacity and 4) reducing exhaled carbon mono-oxide

## Plan for research

### Trial design

Single-blinded, randomized, controlled, parallel group superiority trial with a 1:1 allocation ratio.

### Sample size estimation

Sample size was calculated from g power software as 100 (yoga=50; control=50) where effect size is taken as 0.59 from a study with similar parameters ( Jeffries *et al.*, 2020) by fixing α (0.05) and power of the study as (0.95) considering drop out of 30%

### Study setting

**Figure 1.**
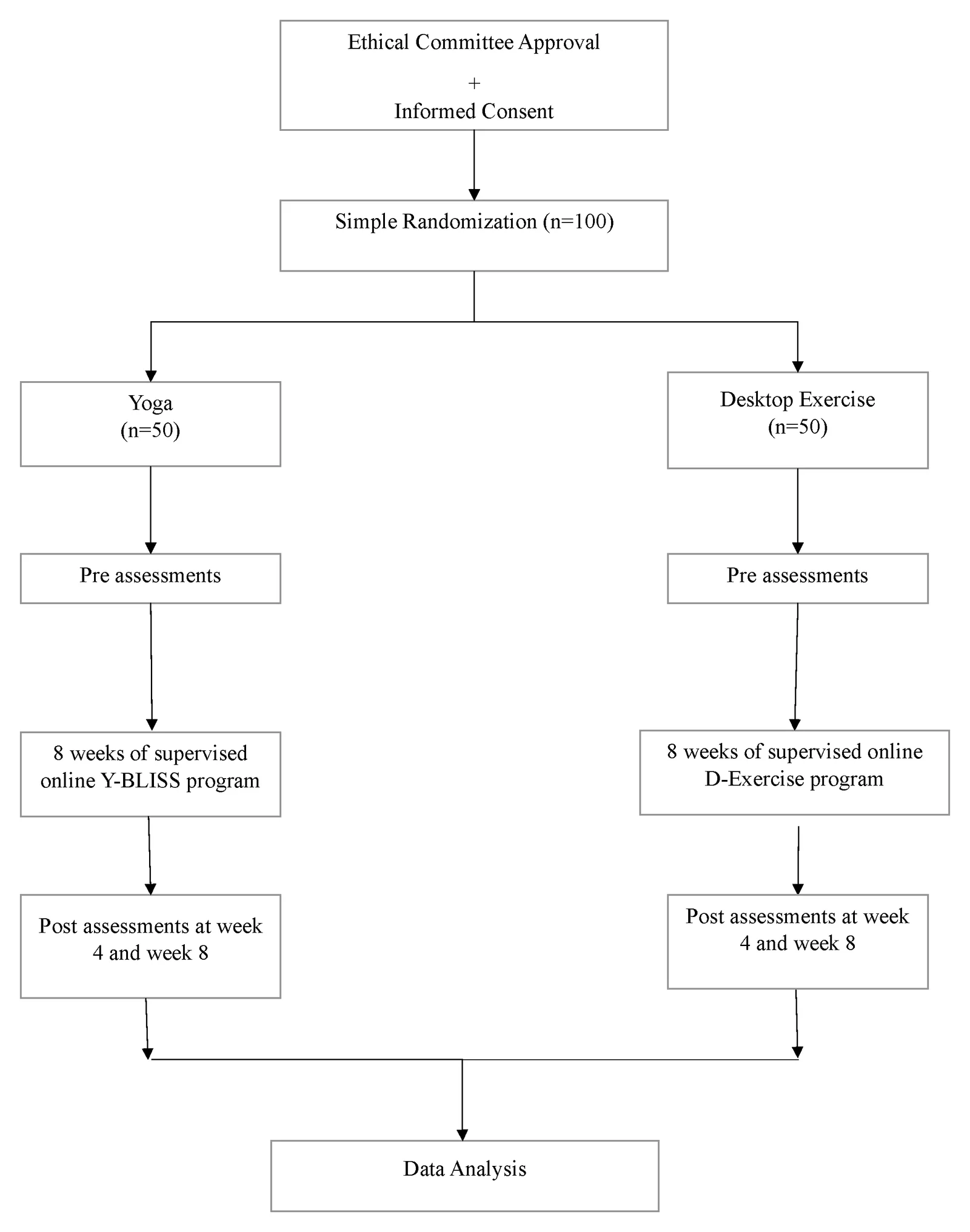
Study design outline.

Participants with at least mild severity of tobacco use disorder (dependence on cigarette smoking with FTND scores above 3) availing the outpatient/ inpatient clinical services under the Centre for Addiction Medicine, Department of Psychiatry, and Department of Integrative Medicine, National Institute of Mental Health and Neurosciences (NIMHANS), Bengaluru, India will be approached. Participants will also be screened in the NIMHANS Centre for Well-being and online social media platforms (through an advertisement of the research project). The course of the trial, its benefits, possible demerits, voluntary participation with a choice to quit the study at any time will be explained in simple comprehensible language. Patients satisfying the inclusion criteria will be requested to provide written informed consent. Consecutive consenting patients will be randomly assigned to one of the two groups – Standard treatment + Yoga-breath linked intervention to stop smoking (Y-BLISS arm), or Standard treatment + Desktop Exercises (D-exercise arm) alone by the biostatistician. Allocation sequence will be generated by biostatistician using computer-generated random numbers and allocation concealment will be done using opaque sealed envelopes. A validated and standardized Y-BLISS program will be used as an intervention. Although double blinding will not be possible in the study, the clinicians treating the subjects, the outcome assessors, and data analysts will remain blind to the subject-group allocation status throughout the period of the study. All participants will be continuing their routine standard care (Nicotine replacement therapy and psycho-social support) till the study concludes. Figure 1 provides the study design outline. Nicotine dosage will be recorded at the beginning of trail; dosage will be adjusted based on the clinical presentation of the patient by the treating psychiatrist.

### Selection of participants

***Inclusion criteria***

The following criteria shall be used to include the subjects for the study

a) Volunteers above 18–55 years of age

b) All gender

c) No prior exposure to yoga

d) Those with FTND scores above 3

***Exclusion criteria***

The following criteria shall be taken into consideration to exclude subjects

a) Those who underwent any recent surgery in the past 6 months.

b) Those with very low level of dependence (FTND of 3 or below)

c) Currently enrolled in a structured CBT or Quit-Smoking program

d) Two or more sessions of yoga in the last one month.

e) Medical conditions that might make participation of Yoga difficult or potentially hazardous (e.g., heart failure, ischemia, and hypertension).

f) Subjects with neurodegenerative disorders and neurological disorders like stroke,

g) Major depressive disorders – psychiatric illness, anxiety disorder, cognitive impairment, severe hearing and visual impairments

h) Pregnant women

i) Those with co-morbid dependence on other substances except alcohol

j) Those with severe alcohol dependence or in those in the state of complicated withdrawal

### Clinical assessment

The demographic (age, gender, height, weight, education level, income, marital status, BMI, co-morbid medical conditions and psychiatric illness) and clinical information will be collected using structured scales and proforma. Previous quit attempts will be noted, readiness to quit, confidence to quit, motivation to quit will be recorded (Motivation to quit smoking, Confidence in quitting, and Readiness to quit using three items answered on a 10-point Likert scale). The data regarding average number of cigarettes smoked per day and number of yoga sessions practiced per day data will be collected on weekly basis from the diary of the same maintained by the participants.

Motivation To Stop Smoking (MTSS) scale consists of 7 item questionnaires, effectively combines both current desire and intention to stop smoking – two key components of motivation into one single response scale, ranging from 1 (lowest) to 7 (highest level of motivation to stop smoking) ( Smit *et al.*, 2011a). The scale consists of 2 item questionnaires, which measures in the Heaviness of Smoking Index (HSI), time to first cigarette of the day (TTFC) and daily consumption (cigarettes per day [CPD]). A peak flow meter is a portable, easy-to-use device that measures lung capacity how well the lungs are able to expel air. Recent tobacco use, responding to cessation and reduced use with tobacco is measured using CO analyser machine. Table 1 provides the schedule of assessment.

**Table 1.** Schedule of assessments.

Assessments	Baseline	4 ^th^ Week	8 ^th^ Week
**Clinical assessment**	P	P	P
Heaviness of Smoking Index	P	P	P
Fagerstrom Test for Nicotine Dependence	P	P	P
Peak Flow Meter (Liters per minute (L/min))	P	P	P
Exhaled CO (parts per million (ppm))	P	P	P
Cigarette Per Day	P	P	P
Motivation To Stop Smoking Scale	P		
Readiness to Quit	P		
Confidence to Quit	P		
Motivation to Quit	P		
BMI(Kg/m) ^2^	P	P	P
Constipation Symptoms (VAS)	P		
Previous Smoking Quit Attempts	P		
Maximum Days of Abstinence	P		
Average Number of cigarettes per day	P		
Age of first exposure	P		
Family History of Smoking	P		

### Data storage, handling, and statistical analysis

A data monitoring committee (DMC) will be created, which will be chaired by the biostatistician. The DMC will be independent from the sponsor and any competing interests. Data handling, coding and password-protected data storage will be supervised by a biostatistician. The biostatistician will also supervise data anonymization and data analysis. The data will be suitably anonymized so that no identification features are revealed. Data anonymization will be done by the biostatistician creating a mirror image of a database and implementing alteration strategies, such as character shuffling or encryption.

### Data analysis

The R programming language (R version 4.4.1) will be used to analyse the data ( R Core Team, 2022; https://www.r-project.org/). Details of dropped-out/withdrawn patients will be recorded. Missing data, drop-outs, non-compliance, and adverse events will be managed in collaboration with the biostatistician and supervisor. The data of all randomized participants will be analysed using intent-to-treat analysis and mixed model multivariate analysis. Information regarding solicited and spontaneously reported adverse events and other unintended effects of trial interventions or trial conduct will be documented and reported to the DMC as well as the ethics committee of the institution. The DMC will have access to interim analysis results and will take the decision to terminate the trial in case of unreasonable morbidities or mortalities among the recruited participants during the trial. Information obtained from the interim analysis may be used for enhancing trial efficiency by updating design and/or sample size adjustments, this will be done as per the suggestions of DMC and after due approval from the funding agency. Trial conduct will be audited once in a year by the DMC, the process will be independent from investigators and the sponsor.

### Intervention

The yoga sessions would be supervised by a certified yoga therapist. Details regarding Y-BLISS intervention is provided in Table 2.

**Table 2.** 12-minute Y-BLISS Program details.

SL.NO.	Practice	Number of cycles	Duration (minutes)
1	Shighra Vishranti Kriya - Instant Relaxation Technique (IRT)	1	1
2	Shvasa Kriya - Deep Belly Breathing (Inhale:Exhale) (6:4:8:4)	5	2
3	Kapalabhati -Forehead Shining Breath	1 cycle 40 strokes	1
4	Bahya Kumbhaka in Dhyana Mudra–External retention of breath	1	1
5	A-U-M Chanting in a single breath (ratio 1:1:1)	5	2
6	Nadi Shuddhi / Alternate nostril breathing (with mental mantra chant)	6	3
7	Breath awareness in Dhyaan mudra – “Breath in - I am not the body; Breath out – I am not the mind” Positive affirmation in deep silence - “I am happy, healthy and satisfied.”		2

The participants will be taught a 12-minute Y-BLISS program every day in the morning before breakfast for 5 days a week for 8 weeks via an online video-conferencing platform. Initial one week (5 sessions) will be required to train the participants in the program after that a video along with a YouTube link ( https://youtu.be/L6mN9GOXrbY) of the program will be shared with the participants and they will be asked to practice the program as and when required whenever they feel excessive craving.

### Control group

Participants will be taught Desktop exercise (D-exercise) program every morning. The delivery of the D-exercise program will match that of Y-BLISS program in all the aspects related to the mode of delivery, duration, therapist interaction, content, flow and follow-up of the program. Table 3 provides the required details.

**Table 3.** 12-minute desktop exercise (D-exercise) module.

Desktop Exercises Module			
Sl No	Stretches	No of Cycles	Duration
1	Neck Rolls	5 Round	30 sec
2	Shoulder Rolls	5 Round	30 sec
3	Chest Stretch	5 Round	30 sec
4	Upper Back Stretch	5 Round	30 sec
5	Hamstring Stretch	5 Round	30 sec
6	Bent-Knee Stretch	5 Round	30 sec
	Core		
1	Seated Bicycle Crunches	5 Round	1 Min
2	Lower-Abs Leg Lifts / one and two legs	5 Round	1 Min
3	Oblique Twists	5 Round	1 Min
	Upper Body		
1	Arm Circles	5 Round	30 sec
2	Desk Push-Ups/Wall Push-Ups	5 Round	30 sec
3	Arm Pulses		
	Lower Body		
1	Calf Raises	5 Round	30 sec
2	Wall Sits	5 Round	30 sec
3	Lunge	5 Round	30 sec
4	Chair Squats	5 Round	30 sec
	Cooling down		
1	Sit relaxed with closed eyes and consciously try to relax body muscles from head to toe		3 minutes

## Description of some major assessments

### Motivation To Stop Smoking (MTSS) scale

The scale consists of 7 item questionnaires, effectively combines both current desire and intention to stop smoking – two key components of motivation into one single response scale, ranging from 1 (lowest) to 7 (highest level of motivation to stop smoking) ( Smit *et al.*, 2011b).

### Heaviness of Smoking Index (HSI) / Smoking rate

The scale consists of a 2-item questionnaire. It measures the Heaviness of Smoking Index (HSI), time to first cigarette of the day (TTFC) and daily consumption (cigarettes per day [CPD]). These are strong predictors of quitting behavior ( Borland *et al.*, 2010).

### Fagerstrom Test for Nicotine Dependence [FTND]

The Fagerström Test for Nicotine Dependence contains six items to evaluate quantity of cigarette consumption, dependence and compsulsion to use. It is a standardized tool used extensively in research related ti nicotine dependence. Higher score on the scale indicates higher dependence on nicotine ( Heatherton *et al.*, 1991).

### Peak flow meter

This device measures the force of exhaled air in liters per minute. It is an indicator of lung functions. This device is portable, easy to use and scientifically validated ( Dekker *et al.*, 1992).

### Exhaled carbon monoxide

CO biomarker is well-established with recent tobacco use and to distinguish tobacco users from nonusers, responding to cessation and reduced use, and having a dose-response with tobacco use. CO analyzer machines calculate CO in ppm in expired air. Some CO analyzer machines automatically calculate the Carboxyhemoglobin percentage based on the levels of CO in the expired air. Interpretation CO levels in parts per million is 1–6 is minimal exposure, 7–10 is Second hand, exposure-11 or more is Regular smoker ( Saloojee & Russell, 1980)

## Ethics statement

The proposed study has been approved by the Institutional Ethics Committee (RES/IEC-SVYASA/255/2022 dated 17 ^th^ August 2022) and registered with CTRI (REF/2022/10/059103), date of registration: 14 ^th^ December 2022. Written Informed consent will be taken from all participants by the investigator, and study records will be kept confidential. Subject identifying information will be replaced with codes. Major amendments to the protocol (changes to eligibility criteria, outcomes, analyses) will be communicated to investigators, institutional ethics committee, and trial registries. The study protocol has been prepared using SPIRIT checklist and the trial has been registered under the centre for open science with registration number: **https://osf.io/p2a4w**

## Compensation for loss of time/wages

No Compensation provided

### Implications

This prospective randomized trial will attempt to replicate and confirm the utility of Yoga in the clinical management of TUD, which has worldwide relevance. The study may also help establish a neurobiological basis for the effects of Yoga in substance use disorders.

### Plans for data management, sharing and dissemination of research findings

All information pertaining to the participants would be kept confidential under the researchers. For conducting the study, each participant would be number coded instead of their name which would be only available to the researchers. Barring the Principal investigator and the study coordinator, none will have access to the final trial dataset.

## Roles and responsibilities

HB, CS, JM and BH designed the study. HB, JM VLN, and BH will help in patient recruitment. HB, BH, AS and EM will supervise assessments. NK, KS and CS will help in training the yoga therapist who will deliver the intervention. CS will help in developing a digital platform for delivering the yoga program. BH will supervise randomization, data management and statistical analysis. 

## Dissemination policy

The findings obtained from the study will be shared with participants. As mentioned above no confidential information will be shared. Data will be published in a peer-reviewed scientific journal along with presentations at conferences for spreading and discussing the findings of the study.

## Ethics and consent

The proposed study has been approved by the Institutional Ethics Committee (RES/IEC-SVYASA/255/2022 dated 17 ^th^ August 2022) and registered with CTRI (CTRI/2022/12/048117), date of registration: 14 ^th^ December 2022. Written Informed consent will be taken from all participants by the investigator.

## Data Availability

No data are associated with this article. OSF: SPIRIT checklist and flow chart for ‘Efficacy of yoga-breath linked intervention to stop smoking (Y-BLISS) in reducing number of cigarettes smoked per day among smokers attempting to reduce or quit: Study protocol for a randomized controlled trial’. https://doi.org/10.17605/OSF.IO/P2A4W ( Chandra Shekar *et al.*, 2024). Data are available under the terms of the Creative Commons Attribution 4.0 International license (CC-BY 4.0).

## References

[ref-1] Allen AM Oncken C Hatsukami D : Women and smoking: the effect of gender on the epidemiology, health effects, and cessation of smoking. *Curr Addict Rep.* 2014;1(1):53–60. 10.1007/S40429-013-0003-6 27213132 PMC4871621

[ref-3] Borland R Yong HH O’Connor RJ : The reliability and predictive validity of the heaviness of smoking index and its two components: findings from the international tobacco control four country study. *Nicotine Tob Res.* 2010;12(Suppl 1):S45–50. 10.1093/NTR/NTQ038 20889480 PMC3307335

[ref-4] Cohen S Lichtenstein E : Perceived stress, quitting smoking, and smoking relapse. *Health Psychol.* 1990;9(4):466–478. 10.1037/0278-6133.9.4.466 2373070

[ref-5] R Core Team: R: a language and environment for statistical computing. R Foundation for Statistical Computing, Vienna, Austria, 2022;2013. Reference Source

[ref-6] Dekker FW Schrier AC Sterk PJ : Validity of peak expiratory flow measurement in assessing reversibility of airflow obstruction. *Thorax.* 1992;47(3):162–166. 10.1136/thx.47.3.162 1519192 PMC1021004

[ref-8] Global Adult Tobacco Survey [GATS]. 2016; [Last accessed date on 2022 Nov 29]. Reference Source

[ref-2] Gupta R Bhatt G Singh R : Enforcement of COTPA in India- current status, challenges and solutions. *Indian J Tuberc.* 2024. 10.1016/j.ijtb.2024.06.007 39890380

[ref-9] Heatherton TF Kozlowski LT Frecker RC : The fagerström test for nicotine dependence: a revision of the fagerström tolerance questionnaire. *Br J Addict.* 1991;86(9):1119–1127. 10.1111/j.1360-0443.1991.tb01879.x 1932883

[ref-10] Jeffries ER Zvolensky MJ Buckner JD : The acute impact of hatha yoga on craving among smokers attempting to reduce or quit. *Nicotine Tob Res.* 2020;22(3):446–451. 10.1093/ntr/nty263 30551146

[ref-20] Jha P Chaloupka FJ Moore J : Tobacco addiction. *Disease Control Priorities in Developing Countries.* 2nd edition.2006. 21250321

[ref-11] Jha RK Li PH : Effects of alternate nostril breathing on quitting smoking. *The International Journal of Science Inventions Today (IJSIT).* 2017. Reference Source

[ref-12] Khanna S Greeson JM : A Narrative review of yoga and mindfulness as complementary therapies for addiction. *Complement Ther Med.* 2013;21(3):244–52. 10.1016/j.ctim.2013.01.008 23642957 PMC3646290

[ref-13] Koçak ND Eren A Boga S : Relapse rate and factors related to relapse in a 1-year follow-up of subjects participating in a smoking cessation program. *Respir Care.* 2015;60(12):1796–1803. 10.4187/respcare.03883 26286738

[ref-14] Chandra Shekar MV Holla B Mahadevan J : Efficacy of Yoga-Breath Linked Intervention to Stop Smoking (Y-BLISS) in reducing number of cigarettes smoked per day among smokers attempting to reduce or quit: study protocol for a randomized controlled trial.2024. 10.17605/OSF.IO/P2A4W

[ref-19] Narasimha VL Mathew Y Anil S : A study to evaluate availability of tobacco cessation services in Bengaluru, India. *Asian J Psychiatr.* 2021;58: 102600. 10.1016/j.ajp.2021.102600 33607349

[ref-15] National Family Health Survey - NFHS-5 (|y2019-21)-Compendium of factsheets, Ministry of Health and Family Welfare, Government of India, 2019.2021; [Last accessed on 2022 Nov 29]. Reference Source

[ref-21] Pradeepkumar PC Murthy P Lohit RP : Impact of Covid-19 on caller characteristics and quit rates: experience of regional tobacco quitline from India. *Nicotine Tob Res.* 2023;25(2):247–53. 10.1093/ntr/ntac013 35023566 PMC8807263

[ref-16] Reitsma MB Kendrick PJ Ababneh E : Spatial, temporal, and demographic patterns in prevalence of smoking tobacco use and attributable disease burden in 204 countries and territories, 1990–2019: a systematic analysis from the global burden of disease study 2019. *Lancet.* 2021;397(10292):2337–2360. 10.1016/S0140-6736(21)01169-7 34051883 PMC8223261

[ref-17] Saloojee Y Russell MAH : Expired air carbon monoxide: a simple breath test of tobacco smoke intake. *Br Med J.* 1980;281(6238):484–485. 10.1136/BMJ.281.6238.484 7427332 PMC1713376

[ref-7] Shiffman S Hickcox M Paty JA : Progression from a smoking lapse to relapse: prediction from abstinence violation effects, nicotine dependence, and lapse characteristics. *J Consult Clin Psychol.* 1996;64(5):993–1002. 10.1037//0022-006x.64.5.993 8916628

[ref-18] Smit ES Fidler JA West R : The role of desire, duty and intention in predicting attempts to quit smoking. *Addiction.* 2011;106(4):844–851. 10.1111/j.1360-0443.2010.03317.x 21205048

