# Supplementary material for: Efficacy of yoga-breath linked intervention to stop smoking (Y-BLISS) in reducing the number of cigarettes smoked per day among smokers attempting to reduce or quit: Study protocol for a randomized controlled trial
Source: Wellcome Open Res. 2026 Aug 11;9:693. Originally published 2024 Nov 26. [Version 3] doi: 10.12688/wellcomeopenres.22839.3 (PMC13560800; doi:10.12688/wellcomeopenres.22839.3)
Supplement: Peer review history

# Reviewer report: Reviewer response for version 3

**DOI:** 10.21956/wellcomeopenres.30175.r164039

**Published:** 2026-08-20

**Authors:** Laura Tolbanoas

**Affiliations:** 1 University of La Laguna, San Cristóbal de La Laguna, Spain

I have no further comments. I am satisfied with the revisions and the authors' responses.

Is the study design appropriate for the research question?

Yes

Is the rationale for, and objectives of, the study clearly described?

Yes

Are sufficient details of the methods provided to allow replication by others?

Partly

Are the datasets clearly presented in a useable and accessible format?

Not applicable

Reviewer Expertise:

Health Psychology; Yoga-Based Interventions; Mental Health.

I confirm that I have read this submission and believe that I have an appropriate level of expertise to confirm that it is of an acceptable scientific standard.

# Reviewer report: Reviewer response for version 2

**DOI:** 10.21956/wellcomeopenres.29520.r157699

**Published:** 2026-06-22

**Authors:** Laura Tolbanoas

**Affiliations:** 1 University of La Laguna, San Cristóbal de La Laguna, Spain

Summary

 This manuscript presents a protocol for a randomized controlled trial evaluating an innovative adjunctive intervention for smoking cessation among individuals with tobacco use disorder. The study addresses an important clinical and public health problem and proposes a methodologically rigorous design. Strengths of the protocol include prospective registration, ethical approval, random allocation, the use of an active control condition to reduce nonspecific intervention effects, assessor blinding, and the inclusion of objective outcome measures. Overall, the manuscript is clearly written and provides substantial information regarding study procedures. The protocol contains considerable methodological detail; however, several aspects require further clarification to ensure full reproducibility and facilitate interpretation of future findings.

 The following points should be addressed:

 1- Definition of standard treatment. The description of standard treatment requires additional detail, particularly regarding psychosocial support.  Because these components may independently influence smoking cessation outcomes, clearer reporting is necessary to allow replication and interpretation of treatment effects.

 2- Potential confounding due to variation in nicotine replacement therapy. Participants may receive individualized nicotine replacement therapy (NRT) doses determined by treating psychiatrists. While clinically appropriate, variability in NRT exposure may act as a confounding factor.

 The authors should clarify:

 • Whether NRT dosage and treatment modifications will be systematically recorded throughout the study.

 • Whether cumulative NRT exposure or dose changes will be incorporated into statistical analyses as covariates or sensitivity analyses.

 3- Adherence monitoring. The effectiveness of the intervention will depend substantially on participant adherence. The protocol should clearly describe how adherence to both the Y-BLISS intervention and associated desktop exercises will be measured, quantified, and reported. Clear adherence metrics are important for replication and interpretation of efficacy outcomes.

 4- Follow-up duration. The planned follow-up period of eight weeks is relatively short for smoking cessation research, given the risk of relapse after initial abstinence. This may limit the ability to draw conclusions regarding the long-term effectiveness of the intervention. The authors should acknowledge this limitation more explicitly and, if feasible, consider including longer-term follow-up assessments (e.g., at 3, 6, or 12 months).

 5- External validity and exclusion criteria. The exclusion of participants with major depressive disorder, anxiety disorders, and cognitive impairment may limit generalizability because these conditions are highly prevalent among individuals with tobacco use disorder. The rationale for these exclusions should be more thoroughly justified, and the implications for external validity should be discussed.

 Minor Comment

 The phrase “sos basis” may not be universally understood by an international readership. Consider replacing it with “on an as-needed basis.”

 Points that should be addressed to ensure scientific soundness

 The following issues should be addressed prior to indexing because they affect methodological transparency, reproducibility, and interpretation of study findings:

 1- Provide a more detailed description of standard treatment, particularly psychosocial support components.

 2- Clarify how nicotine replacement therapy dosage and modifications will be recorded and handled analytically.

 3- Specify procedures and metrics for monitoring and reporting intervention adherence.

 This is a well-conceived, clinically relevant, and methodologically sound study protocol investigating a promising adjunctive intervention for smoking cessation. The randomized controlled design, active comparator, objective outcome measures, and assessor blinding represent important strengths. Addressing the methodological clarifications outlined above would further improve transparency, reproducibility, and interpretation of the eventual study findings.

Is the study design appropriate for the research question?

Yes

Is the rationale for, and objectives of, the study clearly described?

Yes

Are sufficient details of the methods provided to allow replication by others?

Partly

Are the datasets clearly presented in a useable and accessible format?

Not applicable

Reviewer Expertise:

Health Psychology; Yoga-Based Interventions; Mental Health.

I confirm that I have read this submission and believe that I have an appropriate level of expertise to confirm that it is of an acceptable scientific standard, however I have significant reservations, as outlined above.

# Response

**Published:** 2026-07-28

**Authors:** Hemant Bhargav

**Affiliations:** Integrative Medicine, National Institute of Mental Health and Neurosciences, Bengaluru, India, Bengaluru, Karnataka, India

We thank the reviewer for a rigorous and constructive appraisal. All five points, the minor comment, and the three items flagged for indexing have been addressed in version 3.

1. **Definition of standard treatment.** A new subsection, Standard treatment, specifies the three components delivered to both arms under the WHO MPOWER framework and the National Tobacco Control Program guidelines of India: brief motivational counselling of 5 to 10 minutes at each clinic visit by trained addiction psychiatry staff; nicotine replacement therapy as gum (2 mg or 4 mg) or transdermal patch where clinically indicated, prescribed by FTND-graded severity; and referral to structured psychotherapy for significant comorbidity. Standard care is identical across arms and delivered by clinicians independent of the trial team, and receipt of nicotine replacement therapy and concurrent psychotherapy is recorded at baseline and reported by arm.
2. **Variation in nicotine replacement therapy.** Type and dose are recorded at baseline, and any change in dose or in concurrent psychotropic or psychotherapeutic treatment during the trial is logged as a protocol deviation. Baseline nicotine replacement therapy status is compared between arms and reported with the baseline characteristics. We state explicitly that cumulative exposure is not quantified prospectively and that within-trial dose changes are not modelled as time-varying covariates, and we acknowledge the resulting potential for residual confounding under Limitations rather than specify an analysis the data cannot sustain.
3. **Adherence monitoring.** A new subsection, Adherence monitoring, applies identically to both arms. Attendance at each of the 40 supervised online sessions is recorded by the delivering instructor, and as-needed self-practice is captured by weekly SMS-based self-report. Three metrics are reported per arm: mean supervised sessions attended out of 40, mean self-practice sessions, and total exposure. A completer is defined a priori as attendance at 50 percent or more of supervised sessions, that is 20 of 40, together with completion of the week 8 assessment. The unverifiable nature of self-reported practice is acknowledged, and it is treated as a secondary indicator.
4. **Follow-up duration.** We accept this limitation. The assessment schedule spans baseline, week 4 and week 8 and does not extend beyond the intervention period. Limitations now states that, given the concentration of relapse risk in the months after a quit attempt and the field convention of biochemically verified abstinence at six months or longer, the trial supports conclusions regarding end of treatment reduction and short-term point-prevalence abstinence rather than sustained cessation. Assessment at 3, 6 and 12 months lies outside the scope and resourcing of this efficacy trial and is planned as a follow-on study.
5. **External validity and exclusion criteria.** The reviewer is correct that the original wording was broader than intended. Criterion (g) now excludes only severe or unstable psychiatric illness, specifically schizophrenia, bipolar disorder or a current moderate to severe depressive episode, together with cognitive impairment precluding comprehension of instructions and severe sensory impairment; participants with stable, treated depressive or anxiety disorders on unchanged pharmacotherapy for at least 3 months are eligible. The rationale is one of safety and feasibility, since unsupervised as-needed practice requires reliable self-monitoring and active severe illness would confound both adherence and the craving and dependence outcomes. Limitations note that these disorders are considerably more prevalent in tobacco use disorder than in the general population, that the sample retains part of that comorbidity, and that findings should not be extended to active psychosis, bipolar disorder or current severe depression, for which a supervised adaptation would be required.

**Minor comment.**

 "sos basis" has been replaced with "on an as-needed basis". Additional clarifications. Seven-day point-prevalence abstinence has been added as the final secondary outcome, defined as self-reported complete abstinence over the preceding 7 days with biochemical verification by exhaled carbon monoxide at 10 ppm or below, assessed at baseline, week 4 and week 8, with a 6 ppm sensitivity threshold; the 10 ppm criterion is adopted because ambient carbon monoxide in a large metropolitan setting can elevate expired air values independently of active smoking.

 The prior yoga exposure criteria have been made operationally precise, and the weekly record of cigarettes smoked, and sessions practiced is specified as SMS-based self-report.

 We are grateful to the reviewer, whose comments have materially improved the transparency and reproducibility of the protocol, and we hope the revision is now acceptable for indexing.

# Reviewer report: Reviewer response for version 1

**DOI:** 10.21956/wellcomeopenres.25147.r154332

**Published:** 2026-05-13

**Authors:** Kuntal Ghosh

**Affiliations:** 1 Manipur University, Imphal, Manipur, India

The proposed work is well structured. In the title and introduction, the study's nobility is clearly depicted. The aims and objectives are well-formed. In the methodology, the authors have explained their plan of work with scientific competence. Ethical clearance and clinical trial registration support a sound methodology.  Processes of participant recruitment, the administration of the yoga protocol, and the use of measurement tools support the aims and objectives. The data procurement and analysis part is also well explained. Overall, the research plan shows its scientific capability.     

 The proposed work is well structured. In the title and introduction, the study's nobility is clearly depicted. The aims and objectives are well-formed. In the methodology, the authors have explained their plan of work with scientific competence. Ethical clearance and clinical trial registration support a sound methodology.  Processes of participant recruitment, the administration of the yoga protocol, and the use of measurement tools support the aims and objectives. The data procurement and analysis part is also well explained. Overall, the research plan shows its scientific capability.   

Is the study design appropriate for the research question?

Yes

Is the rationale for, and objectives of, the study clearly described?

Yes

Are sufficient details of the methods provided to allow replication by others?

Yes

Are the datasets clearly presented in a useable and accessible format?

Yes

Reviewer Expertise:

Yoga and its therapeutic applications.

I confirm that I have read this submission and believe that I have an appropriate level of expertise to confirm that it is of an acceptable scientific standard.
